# Explainable deep-neural-network supported scheme for tuberculosis detection from chest radiographs

**DOI:** 10.1186/s12880-024-01202-x

**Published:** 2024-02-05

**Authors:** B. Uma Maheswari, Dahlia Sam, Nitin Mittal, Abhishek Sharma, Sandeep Kaur, S. S. Askar, Mohamed Abouhawwash

**Affiliations:** 1 Department of Computer Science and Engineering, St. Joseph’s College of Engineering, OMR, Chennai, Tamilnadu 600119 India; 2 Department of Information Technology, Dr. M.G.R Educational and Research Institute, Periyar E.V.R High Road, Vishwas Nagar, Maduravoyal, Chennai, Tamilnadu 600095 India; 3 University Centre for Research and Development, Chandigarh University, Mohali, Punjab 140413 India; 4 Department of Computer Engineering and Applications, GLA University, Mathura, Uttar Pradesh 281406 India; 5 Department of Computer Engineering & Technology, Guru Nanak Dev University, Amritsar, Punjab 143005 India; 6 Department of Statistics and Operations Research, College of Science, King Saud University, P.O. Box 2455, Riyadh, 11451 Saudi Arabia; 7 Department of Computational Mathematics, Science, and Engineering (CMSE), College of Engineering, Michigan State University, East Lansing, MI 48824 USA; 8 Department of Mathematics, Faculty of Science, Mansoura University, Mansoura, 35516 Egypt

**Keywords:** Deep neural network, Convolution neural network, Explainable models, Tuberculosis diagnosis, Pre-trained model, Class activation maps, LIME explainer

## Abstract

Chest radiographs are examined in typical clinical settings by competent physicians for tuberculosis diagnosis. However, this procedure is time consuming and subjective. Due to the growing usage of machine learning techniques in applied sciences, researchers have begun applying comparable concepts to medical diagnostics, such as tuberculosis screening. In the period of extremely deep neural nets which comprised of hundreds of convolution layers for feature extraction, we create a shallow-CNN for screening of TB condition from Chest X-rays so that the model is able to offer appropriate interpretation for right diagnosis. The suggested model consists of four convolution-maxpooling layers with various hyperparameters that were optimized for optimal performance using a Bayesian optimization technique. The model was reported with a peak classification accuracy, F1-score, sensitivity and specificity of 0.95. In addition, the receiver operating characteristic (ROC) curve for the proposed shallow-CNN showed a peak area under the curve value of 0.976. Moreover, we have employed class activation maps (CAM) and Local Interpretable Model-agnostic Explanations (LIME), explainer systems for assessing the transparency and explainability of the model in comparison to a state-of-the-art pre-trained neural net such as the DenseNet.

## Introduction

Tuberculosis (TB) is a highly infectious lung infection caused by the bacteria Mycobacterium Tuberculosis. The bacteria affect persons of all ages, but is more widespread among those in their middle years. When a person inhales air tainted with TB bacteria, they get infected. These individuals have a 5- to 10% lifetime risk of having the condition [1]. However, those with a weaker immune system, malnutrition, or diabetes are more likely to get tuberculosis [2]. The illness is widespread, and a 2020 WHO factsheet states that new cases are increasing at a rate of 43% in South-East Asia, 25% in Africa, and 18% in the western pacific area [3].

Tuberculosis is diagnosed in a suspected individual by studying his or her clinical history, physical appearance, and chest radiograph. To diagnose chest anomalies, a posterior-anterior chest radiograph is employed. Lesions may occur in any location of the lungs and vary in size, shape, density, and cavitation. A posterior-anterior (PA) chest radiograph is used to determine the presence of chest abnormalities [4].

Chest radiographs are checked in typical clinical settings by skilled doctors for the identification of tuberculosis. This method, however, is time intensive and subjective. Subjective discrepancies in radiographic illness diagnosis are unavoidable. Notably, chest radiographs of TB are often misclassified as other illnesses with similar radiologic patterns, resulting in patients receiving the incorrect medicine and deteriorating their health state [5]. Additionally, resource-constrained nations, particularly rural ones, have a shortage of qualified radiologists [6].

Due to the scarcity of skilled radiologists for tuberculosis screening and the buildup of massive data sets from low-cost chest radiographs, computer-aided screening has emerged as a feasible option. The widespread use of machine learning techniques in applied sciences has prompted researchers to apply similar ideas to medical diagnostics, such as tuberculosis screening. The Convolution Neural Network (CNN) is a well-researched and proven deep learning model for image classification applications using publicly accessible picture datasets such as IMAGENET. Recently, a variety of CNN-based image classification models, including VGG, ResNet, DenseNet, and MobileNet, have been created for general image classification and also adapted to identify specialty pictures such as Chest X-rays [7–11]. This is accomplished through the use of transfer learning, which entails modifying the top-level layers to train on the specific X-ray images that contain spatial information while retaining the low-level layers that are capable of discriminating more common image textural features such as edges and contours.

Using CNN-based deep learning techniques, computer-aided TB screening has been shown to classify test radiographs with high accuracy. However, there are some limitations to the high accuracy of complex and deeper CNN models (models with numerous convolution layers) [12]. Given their model-agnostic nature, deep CNN models are frequently challenging to interpret. Due to the black-box nature of the models, clinical applications of AI-based Computer Aided Diagnosis (CAD) are still in their nascent stage. As a result, these methods have not yet gained the trust of physicians. The explainability of CNN models for classifying medical images is a widely discussed subject because it is crucial for radiologists to understand how CNN models arrive at their conclusions.

Building comprehensible CNN-based models for medical image classification, particularly in TB screening from radiographs, has significant advantages. First, it offers details about the feature layers that significantly contribute to the prediction outcome, giving insights into how the developed model functions internally. Therefore, based on the necessary image features of the radiographs, which are biomarkers of the disease, the doctor could verify that the model is functioning as intended. Second, interpreting the model can help determine whether the results are trustworthy, allowing doctors to make informed decisions about the management of the disease or the need for human intervention. Better disease prediction outcomes, shorter analysis times, and more widespread clinical application could result from the ability to interpret and explain the deep learning models for TB.

The degree to which deep learning models are explicable is proportional to the model's complexity. Deeper CNN systems have a tendency to produce more accurate classifications, but their underlying principles are difficult to comprehend. For the development of a CNN-based TB disease screening system, there must be a trade-off between explainability and accuracy.

A shallow Convolution Neural Network (CNN) is a type of neural network architecture that has a smaller number of convolutional and pooling layers compared to deep CNNs. Shallow-CNNs are designed for simpler image classification tasks and have fewer parameters, resulting in faster training and inference times. They offer a balance between computational efficiency and performance. Shallow-CNNs can be advantageous in cases where the complexity of deep CNNs is not necessary, such as when capturing discriminative features from relatively simple images [13]. Typically, a simple and shallow CNN model would suffice for initial disease screenings, such as TB. Hence, in this paper, we aim to develop and validate a simple CNN model with fewer feature layers that provides reliable classification performance as well as robust interpretability. However, developing a simple CNN model with good performance metrics is not a simple task. For optimal performance in TB screening, extensive model optimization is required. Consequently, the aforementioned factors led us to develop an explainable system for tuberculosis, which inspired to the following contributions of this research paper:

(i) For tuberculosis screening, simple and shallow-CNN architecture is presented.
(ii) The proposed model's hyperparameters are fine-tuned using a Bayesian optimization technique to provide accurate differentiation of tuberculosis characteristics in Chest X-rays.
(iii) A comparison is made between the proposed shallow-CNN and DenseNet in terms of classification performance and explainability.

## Related work

Computer aided diagnosis of diseases took a prominent stage with the advancement of deep learning techniques especially for analyzing medical images. Convolution Neural Network (CNN) is a standard method for predicting diseases from images such as Chest X-rays, CT and MRI images. Earlier attempts to employ CNN for tuberculosis detection may be found in Liu et al. work’s which involved changing the topologies of two pre-trained nets, AlexNet and GoogleNet. They reported a classification accuracy of 85.68% when evaluated against a large dataset, exceeding the benchmark approaches at the time [14].

Similarly, Liu and Huang evaluated six pre-trained models for TB detection: DenseNet, NasNet, VGG16, InceptionNet, XceptionNet, and ResNet. They enhanced accuracy by altering the activation function and fine-tuning the training settings. They concluded that the best model for tuberculosis classification is DenseNet [15] which was reported with an accuracy of 0.835.

In addition to chest radiography, tuberculosis is frequently diagnosed by studying sputum smear microscopic pictures. In this regard, Samuel and Kanna created a computer-aided method that captures microscopic sputum smear images and extracts features using a modified Inception V3 Net. Following that, a Support Vector Machine (SVM) classifier was trained on the CNN features for the purpose of classifying tuberculosis (TB). They reported an accuracy of 95.05 % in classification, as well as good sensitivity and specificity scores [16]. Similarly, Kuok et al. compared three distinct CNN architectures for TB classification from sputum smear images: single-CNN, Deep networks of CNN, and ensemble-CNN. It was a large study involving 19000 smear images and concluded that deeper neural networks are more capable of providing dependable classification results even when the dataset is unbalanced [17]. They reported a top detection performance of 86% using their refined region-based CNN.

Recently, hybrid deep learning (DL) models for diagnosing medical disorders in lung X-ray images have been proposed in the literature. Bharati et al. merged a classical CNN network, the VGG, with a Spatial Transformer Network (STN) to improve the detection of lung diseases [18]. In comparison to Vanilla CNN and Capsule Nets, their work had a moderate accuracy of 73 %. In a slightly different technique, Tasci et al. employed a voting mechanism to decide on X-ray image for tuberculosis diagnosis using a series of fine-tuned CNN architectures. They showed high accuracy of 97.5% while testing their findings using two publicly available tuberculosis datasets [19].

In more recent work by Ahmed et al. a study on X-ray image-based pneumonia and tuberculosis was done based on differential diagnosis models. It combines deep CNN features (VGG16 and ResNet18) with hand-crafted LDG features (LBP, DWT, and GLCM). It compares pre- and post-PCA VGG16 and ResNet18-integrated ANN performance [20, 21]. Chandra et al. developed simple shape-based geometrical features, combined handcrafted shape features with statistical texture characteristics, developed an algorithm to detect different TB pathologies, and compared proposed methods to state-of-the-art methodologies [22].

Deep neural networks (DNNs) were demonstrated to perform well on X-ray images when used to classify tuberculosis (TB). However, its impact on clinical trials is not fully recognized by the medical community. The increased performance is offset by the incapacity to grasp these deep learning models. When compared to expert or rule-based systems, deep learning-based image categorization algorithms often lack the requisite interpretability. By using DNN-based classification models, we are able to trade off interpretable components for uninterpretable ones, resulting in increased classification performance through increased abstraction or hidden layers. As a result, its use in mass clinical practice is restricted due to the use of black-box models, which limit the physician's comprehension of how the model generates a decision outcome.

Explainable Artificial Intelligence (XAI) solves the interpretability problem of CNN by employing various types of explainer systems, which frequently involve post-hoc analysis to gain insights about the model. Visual inspection based on saliency maps is a prevalent CNN-based XAI technique used in medical imaging applications. These maps highlight discriminant image regions that are utilized by image classification models. Numerous techniques, such as the visual explanations technique based on guided backpropagation, deconvolution, class activation maps (CAM), local interpretable model-agnostic and attention models, have been reported in the literature [23]. The limited applications of these explainer systems could be expanded to include deep neural networks for tuberculosis screening. The majority of earlier attempts to use visual inspection for TB validation involved pre-trained models such as DenseNet [24]. These are extremely complex and computation-intensive CNN architectures that can be explained through visual maps, but are frequently unnecessary for prescreening of tuberculosis from radiographs. Therefore, the objective of this study is to develop a TB screening system with fewer feature extraction convolution layers that has robust classification performance and does not compromise the interpretability of the model.

## Materials and methods

The purpose of this research study is to develop an efficient and interpretable shallow-CNN model for tuberculosis diagnosis. This is in contrast to previous published work that concentrated on validating deep neural models in order to achieve higher classification scores but was not interpretable [13]. We describe the source and type of the chest X-ray utilized to construct the proposed system in this part, as well as the technique for testing the proposed shallow-CNN and pre-trained net for interpretability using class activation maps (CAM) [25] and LIME [26] methods.

### Dataset

**Fig. 1:**
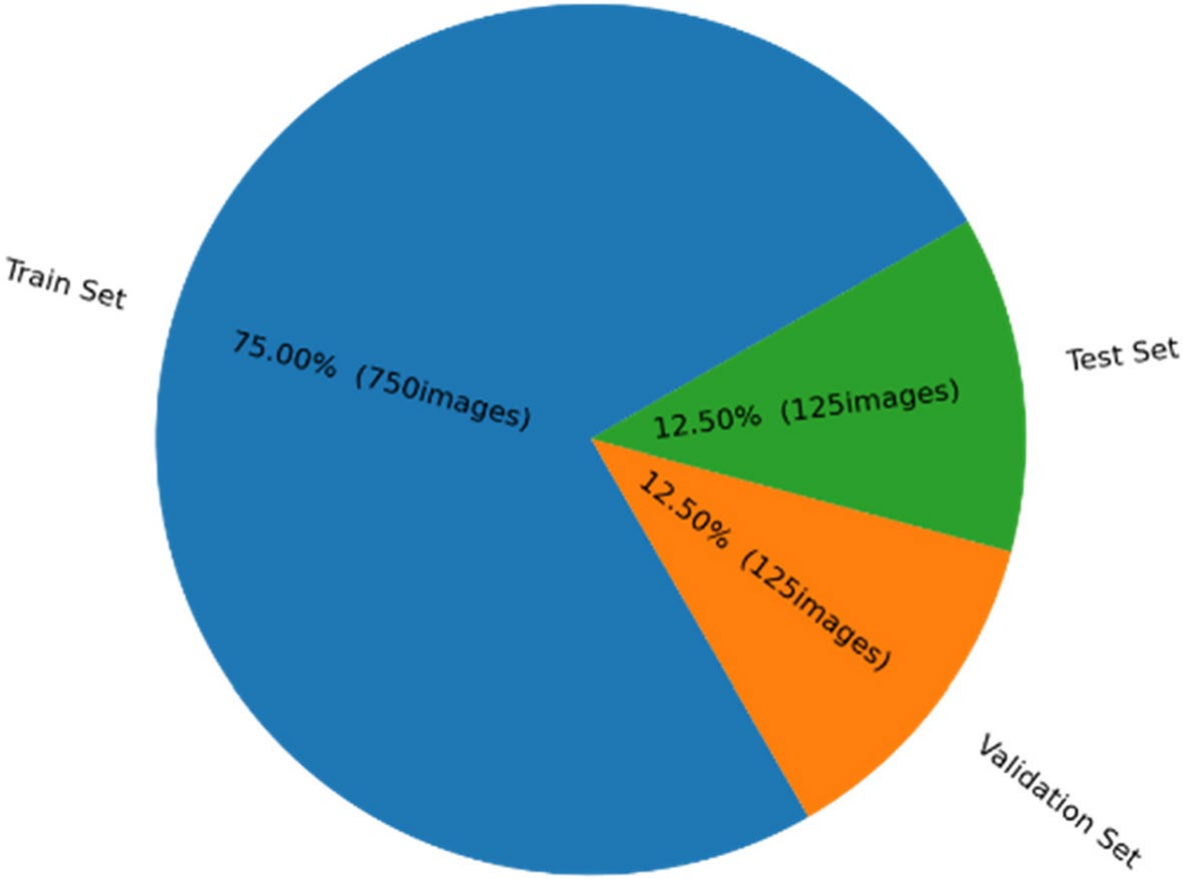
Data split for validating the CNN models used in this study

**Fig. 2:**
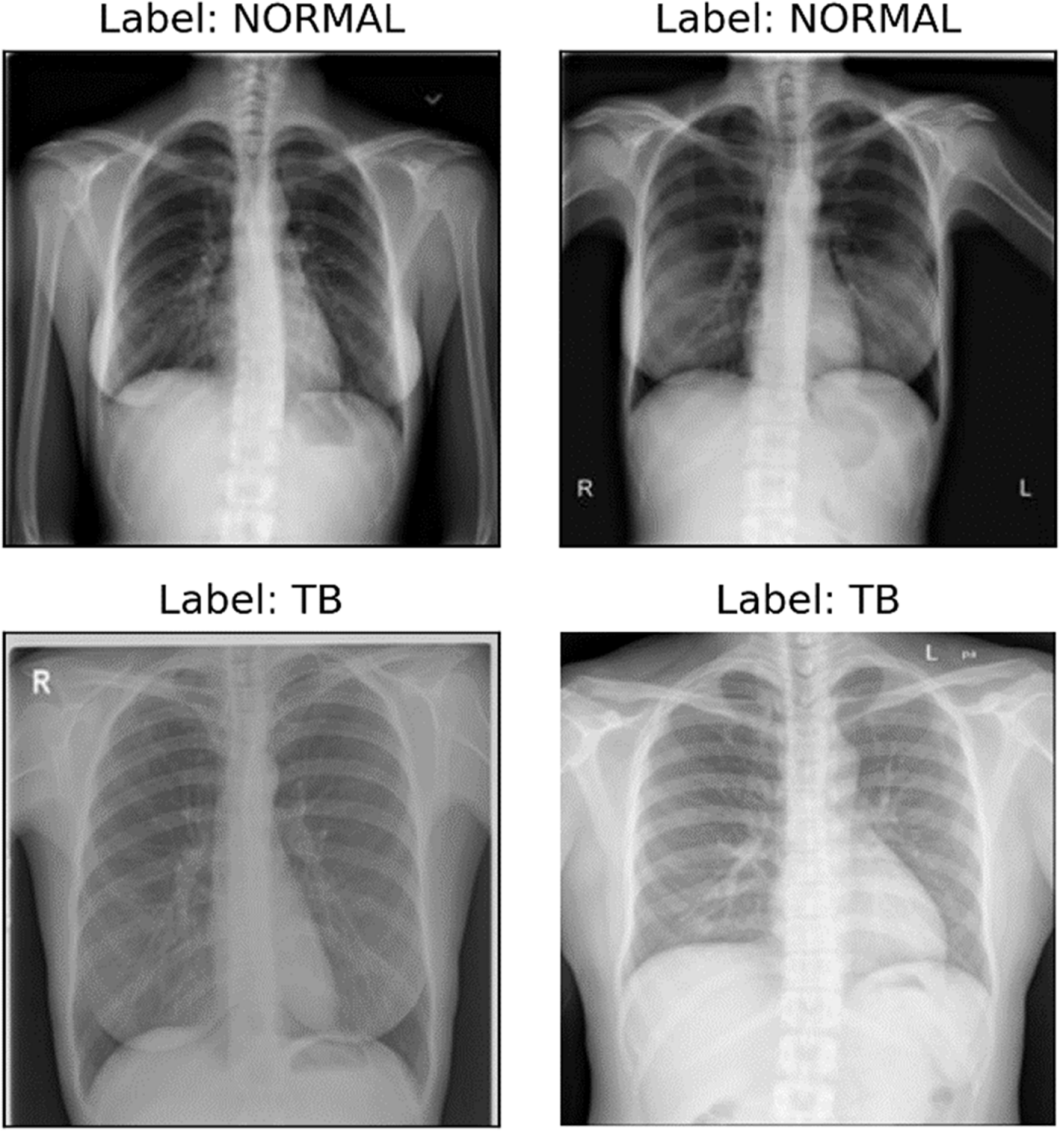
Posterior-Anterior (PA) chest X-ray of the healthy and tuberculosis groups (TB)

The TB chest X-ray used in this investigation were collected from a dataset made freely available by the IEEE Dataport-Tuberculosis (TB) chest X-ray database with the identifier “https://dx.doi.org/10.1109/ACCESS.2020.3031384, 10.21227/mps8-kb56” [6, 27]. We obtained normal and abnormal X-ray images of healthy and TB participants and resized them to 224×224×3 dimensions for use as input to the CNN models. As it is customary to have different dataset for training the model effectively, we divided the available images into three disjoint data groups. We constructed a training set of 750 images, a validation set of 125 images, and a test set of 125 images from these X-ray images, as indicated in Fig. 1 from the aforementioned database. Meanwhile, Fig. 2 illustrates a representative sample of radiographs utilized in this investigation.

In regular clinical study of tuberculosis patients, skilled radiologists visually analyze chest X-ray in order to detect tuberculosis symptoms in the lung areas. For instance, in Fig. 2, the test photographs depicting the lung regions of normal/healthy participants with normal lung function, but the TB images depict some type of pulmonary infiltrates, which may be a symptom of infection. This analysis is a lengthy and laborious procedure that is frequently subjective.

### Implemented scheme

**Fig. 3:**
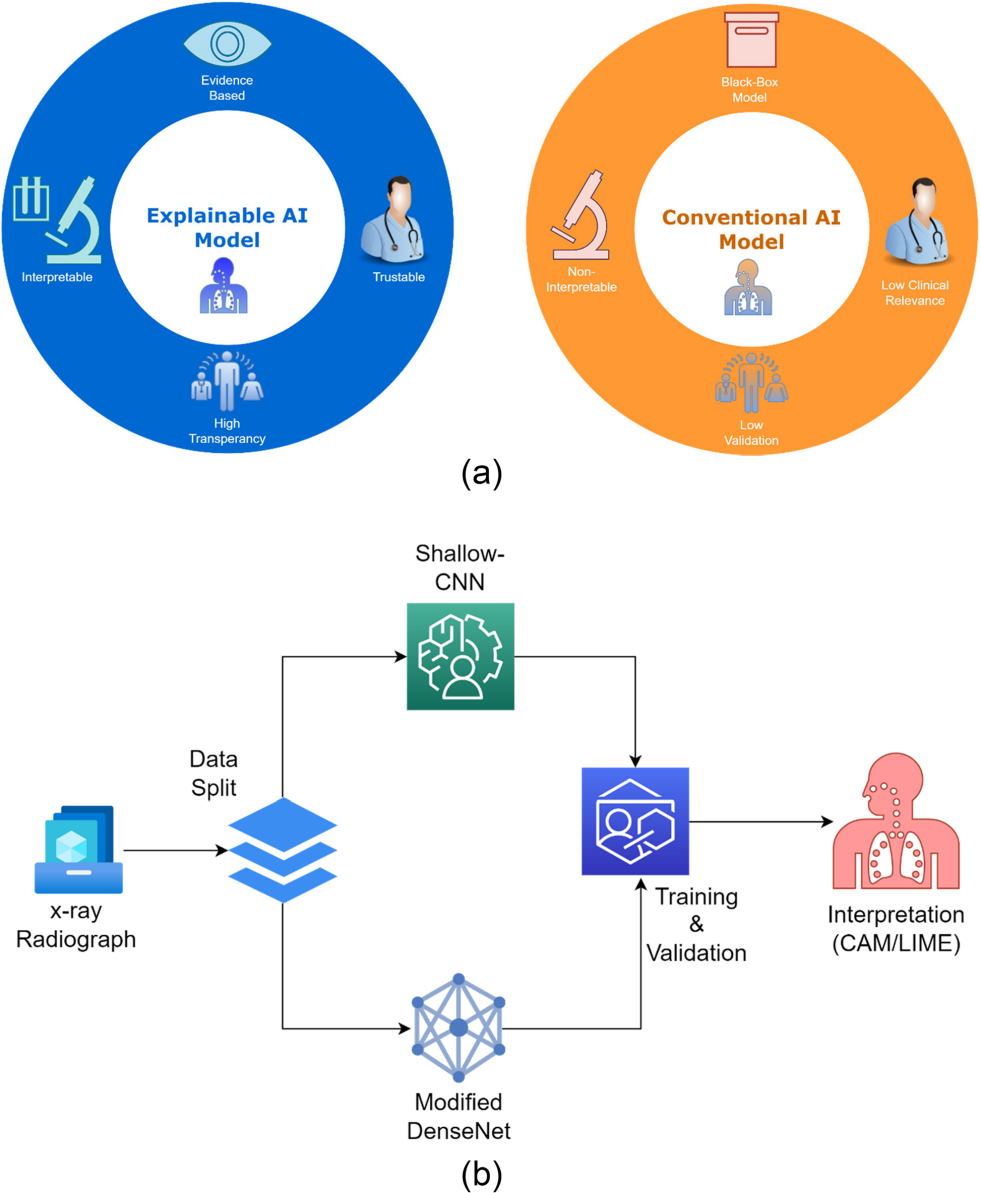
(**a**) Difference between Explainable and Conventional AI Models (**b**) Pipeline for the investigation strategy employed in this article to analyze tuberculosis for explainability

By developing an appropriate CNN model for computer aided tuberculosis diagnosis, the laborious work of visually assessing chest X-ray images for tuberculosis identification is decreased. Along with developing an effective deep learning model, we aim to scrutinize the trained model's class discrimination for proper interpretation of chest anomalies. This is accomplished by visualizing and extracting filter activation maps, as well as generating class activation maps (CAM) to determine which areas are employed by the DL model for class classification of normal and tuberculosis participants, respectively. Figure 3(a) illustrates the difference in approach between explainable and conventional deep learning models in clinical setting. Figure 3 (b) illustrates the proposed approach for verifying DL models for tuberculosis classification.

### Convolution neural nets

CNN is a mathematical abstraction for a shift-invariant hierarchical learning system. It is a collection of specialized neural networks capable of learning complicated spatial patterns directly from the pixel relationships in an image. Unlike traditional models such as support vector machine (SVM) and Random Forest approaches, which extract essential features manually, CNN-based models do the job automatically. This also eliminates any subjectivity that may have been there throughout the feature engineering process. Due to the widespread availability of dedicated hardware such as the graphics processing unit (GPU)/tensor processing unit (TPU), the computational cost of generating CNN activation functions has been rapidly reducing.

With the establishment of the IMAGENET classification competition, which is held on a regular basis to address image classification difficulties, many state-of-the-art models have been found. These included AlexNet, VGGNet, ResNet, InceptionNet, and DenseNet, which all performed very well when classified 1000 classes supplied by IMAGENET [28]. Through a process of transfer learning, the nets were also utilized to solve additional specialized image classification issues in other areas, such as medical image classification. The bottom layers of the models were preserved from their initial training using IMAGENET, but the top layers were changed to understand the intricate spatial patterns associated with the specific picture categorization use case. These pre-trained state-of-the-art CNN models were employed to classify tuberculosis, and high classification accuracy was found. Rahman et al. conducted a comprehensive investigation in which they employed nine such pre-trained models for tuberculosis diagnosis using a transfer learning technique. They demonstrated higher classification performance with the DenseNet on segmented X-ray radiographs [6]. As such, we evaluate the proposed shallow-CNN model and compare its performance and class activation to that of the DenseNet deep learning model in this research study.

### Shallow-CNN for TB detection

Deep neural networks, in particular pre-trained nets based on a transfer learning strategy, are well-known for their high classification accuracy. The precision, on the other hand, comes at the expense of interpretability. In the case of medical image classification, the computer-aided diagnosis system frequently requires the physician or radiologist to provide a transparent and explainable conclusion of the class discrimination for proper interpretation. DNN models are unable of providing any explanations for failure modes. As a result, when it comes to Deep Learning models, there is a trade-off between accuracy and interpretability. To overcome this constraint, we intend to create a simple (fever layers) and interpretable shallow-CNN(S-CNN) model.

A shallow CNN refers to a convolution neural network architecture that has a relatively small number of layers compared to deeper CNN models. It typically consists of only a few convolutional layers followed by pooling and fully connected layers. Shallow CNNs are often used for tasks that require less complex feature extraction, such as image classification or object detection in simpler datasets [29].

**Fig. 4:**
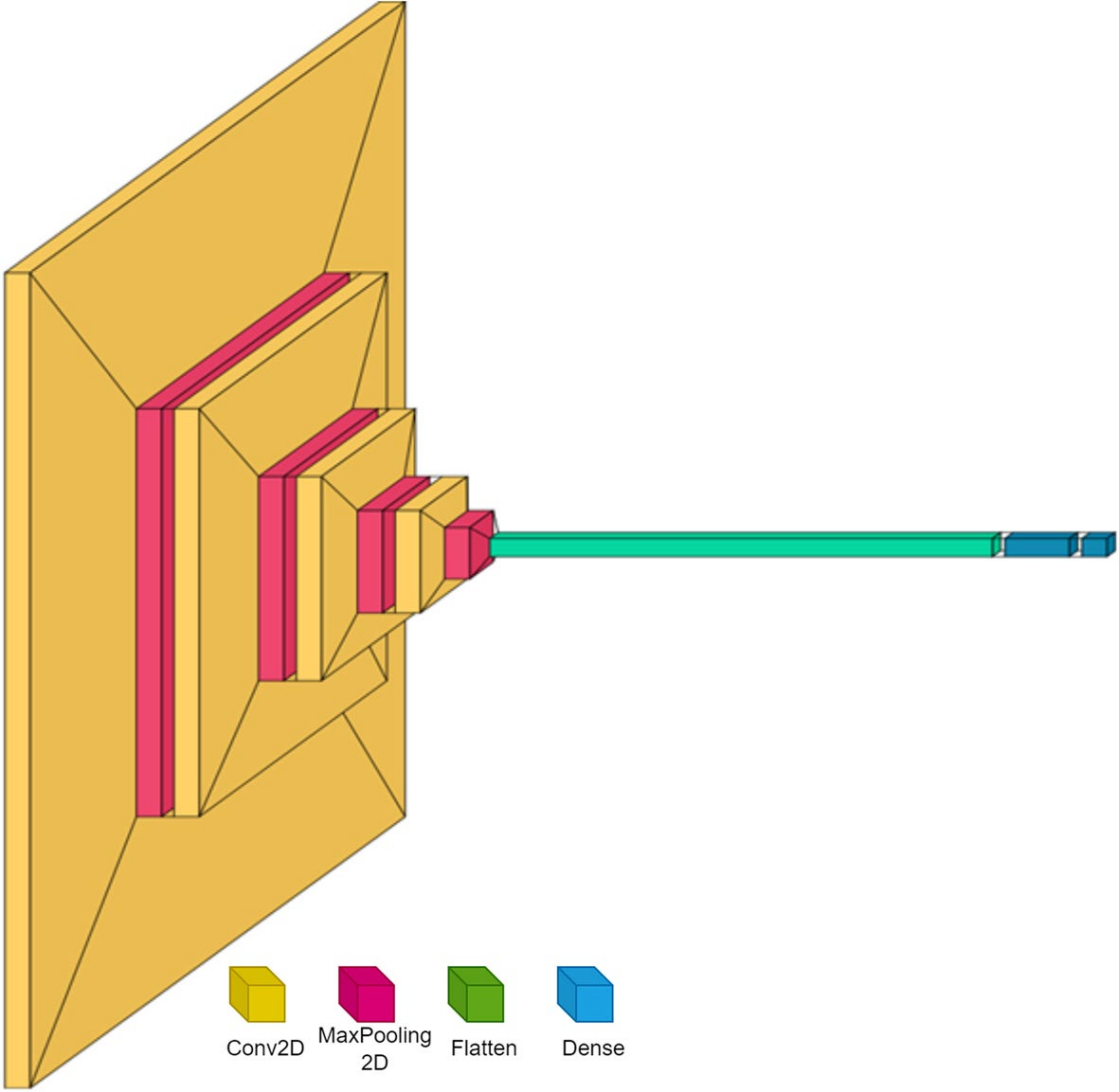
Schematic of the proposed interpretable Shallow-CNN classification algorithm for tuberculosis

S-CNN functions similarly to a deep neural network but has less convolution and dense blocks. The design of the S-CNN for TB screening is based on the nature of the disease's lung manifestations in the affected population. One of the symptoms of tuberculosis is the formation of granuloma complex in the lung's prominent regions. Consequently, the lung portions of the chest X-ray have a significantly different texture than the rest of the image. We believe that S-CNN with fewer layers could be adequate for learning these discriminant radiograph features for TB screening. For this reason, we utilized only four convolution layers and a variable number of kernels with different sizes. Figure 4 depicts the proposed S-CNN for TB screening, which consists of a four-layer convolutional and max-pooling structure followed by two feedforward neural networks.

The architecture of the proposed s-CNN consists of different modules which could be explained as follows:

- Chest X-ray Input Layer: The chest radiographs are of 224×224 dimension with a channel depth of 3.
- Convolution Layer: These are the feature extraction layers that learns the textural features of the radiographs. Based on kernel size different image features are extracted. S-CNN consists of four convolution layers with varying depth for learning TB manifestations. The operation of the layer could be given as:

$${{\text{g}}}_{\theta }\left[m,n\right]=\left({f*h}_{\theta }\right)\left[m,n\right]=\sum_{j}\sum_{k}{h}_{\theta }\left[j,k\right]f\left[m-j, n-k\right]$$

Here, the feature map ${{\text{g}}}_{\theta }$ is obtained by convolving the kernel ${h}_{\theta }$ with the input image $f$. Here ‘m’ and ‘n’ specify the coordinates of the output image and ‘j’ and ‘k’ are the coordinates of the input pixel, relative to the output pixel. By the process of the convolution the network trains and get activated to localized image feature based on the instantiated kernel.

(iii) Pooling Layer: Once the image features are extracted in the Convolution layers, Pooling layer reduces the feature redundancy through a down-sampling process. There are two main advantages of using the pooling layer after the convolution layers namely: (a) the layer reduces the total number of trainable parameters of the model there by increasing the training speed (b) as the number of parameters are reduced, the problem of overfitting of the model to the input vector is limited thus producing good generalization capability to the network. The operation of pooling layer could be given as:

Here, ${s}_{p}$ is the down-sampled feature map and ${T}_{p}$ represents the pooling transform which could be a max, min or average of the input vector.

(d) Dense Layer: It is the fully connected feed-forward neural network with takes the flattened feature maps of the preceding feature extraction layer and computes an overall activation of the network. These are high-level feature extraction layers.
(e) Classification Layer: It is the output layer which performs the outcome of the input image and categorizes the type of image namely whether normal or abnormal radiograph based on SoftMax activation function.

When interpretability and accuracy are of equal importance in the domain of medical image classification, the selection of neural network architecture is critical. Within this particular framework, the Shallow Convolutional Neural Network (S-CNN) emerges as a noteworthy substitute for conventional deep models. In order to attain a thorough comprehension of its merits, we establish a significant analogy with AlexNet [28], an early adopter of deep learning architectures that was initially developed to tackle the ImageNet challenge.

AlexNet is widely recognised for its adaptability and resilience when confronted with intricate image datasets, whereas the S-CNN intentionally strives for simplicity and interpretability in order to attain similar outcomes in the analysis of medical images. This comparative analysis provides valuable insights into the compromises that exist between efficiency and depth, thereby clarifying the contextual appropriateness of each architectural design, with a particular focus on the specialised field of tuberculosis (TB) screening. Although both the Shallow Convolutional Neural Network (S-CNN) and AlexNet aim to classify images, they diverge considerably in terms of structure and intricacy, with each providing unique benefits.

The S-CNN, as its nomenclature implies, intentionally employs a shallow architecture consisting of a limited number of convolutional layers. This design decision is predicated on the notion that a reduced quantity of layers is adequate for distinguishing crucial radiographic characteristics associated with tuberculosis screening. AlexNet, on the other hand, is distinguished by its profound architecture, which comprises numerous dense and convolutional layers. It is capable of acquiring complex hierarchical features from a variety of datasets, such as ImageNet, due to this depth. S-CNN prioritises interpretability and simplicity. The S-CNN utilises fewer convolution layers in order to extract crucial features from chest X-rays, particularly those that are suggestive of tuberculosis manifestations.

AlexNet, renowned for its ability to acquire intricate features by virtue of its more profound architecture, demonstrates exceptional performance on intricate image datasets. However, it might be excessively designed for tasks that solely require feature extraction. The S-CNN architecture is specifically designed for tuberculosis screening and is informed by the unique attributes of TB manifestations observed in chest X-rays. By specialising in this area, one can achieve optimal performance in specific medical imaging tasks.

AlexNet: Initially developed for the classification of general-purpose images, it may be excessively complex for applications such as tuberculosis screening, where a more straightforward model can attain equivalent outcomes with improved interpretability. In essence, the Shallow-CNN and AlexNet exemplify contrasting methodologies in the realm of image classification. The former places emphasis on simplicity, interpretability, and optimization specific to the task at hand. In contrast, the latter, due to its more profound architecture, strives to tackle more extensive image classification tasks by acquiring knowledge of intricate features from intricate datasets. The selection between them is contingent upon the particular requirements and limitations of the given implementation.

### Model tuning

The suggested model's configuration for tuberculosis classification must be extensively tweaked to obtain the best hyperparameters listed in Table 1. This is not straightforward, as there are an infinite number of possible parameter combinations for the shallow-CNN model in order to obtain reliable classification scores. Thus, even though the model has fewer layers, utilizing a grid search strategy is not intuitive and incurs a computational penalty. As a result, we employ a Bayesian optimization strategy to estimate the proposed model's optimal parameters [30, 31].

**Table 1:** Hyperparameter configuration and limits for tuning the shallow-CNN model

**Hyper-parameter**	**Range**
Kernel Size	[3 to 11]
Number of filters in convolution layers	[16 to 128]
Kernel Stride	[1 to 5]
Pooling Method	[MaxPooling, AveragePooling, GlobalMaxPooling]
Number of units in dense layer-1	[128 to 1024]
Learning rate	[0.1 to 0.001]
Optimizer	[Adam, AdaGrad, AdaDelta, SGD]

**Fig. 5:**
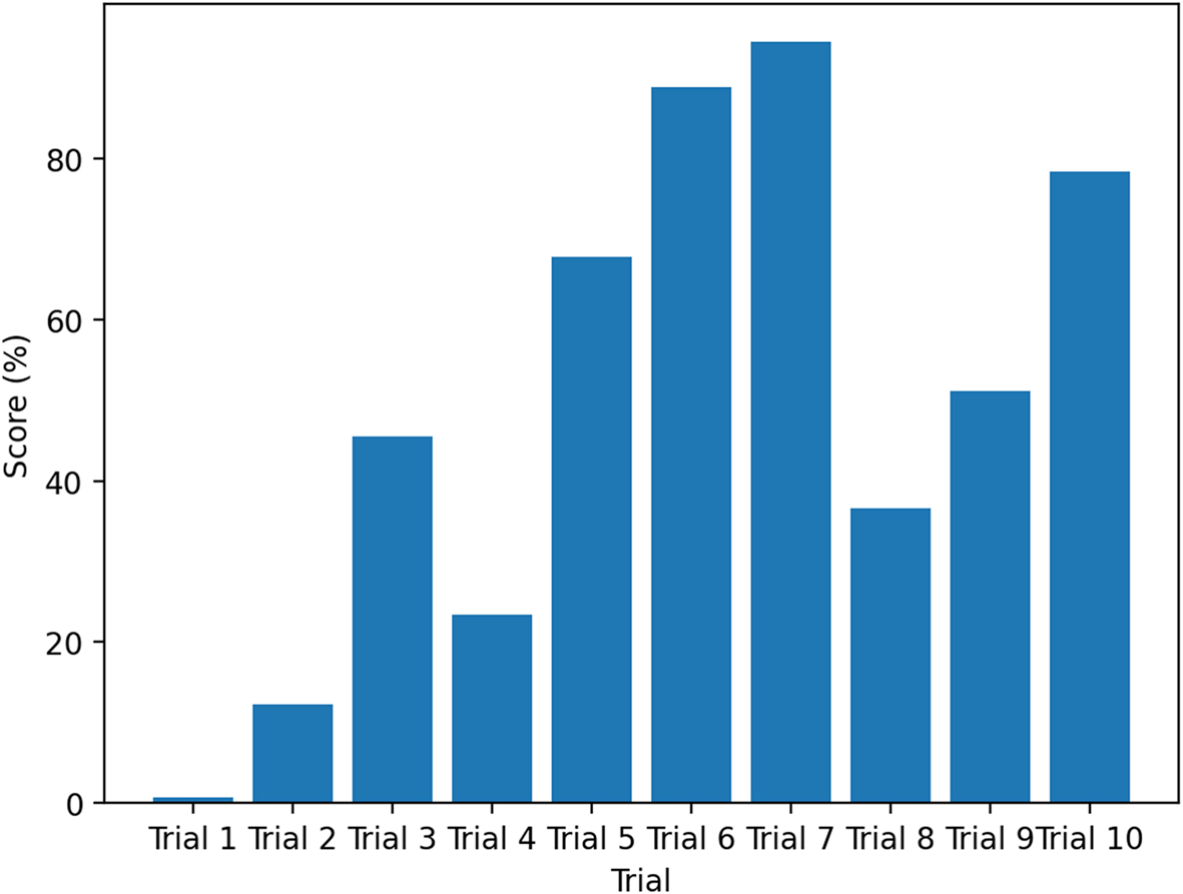
Hyperparameter tuning process for selection of best configuration for the proposed Shallow-CNN TB classification model

**Fig. 6:**
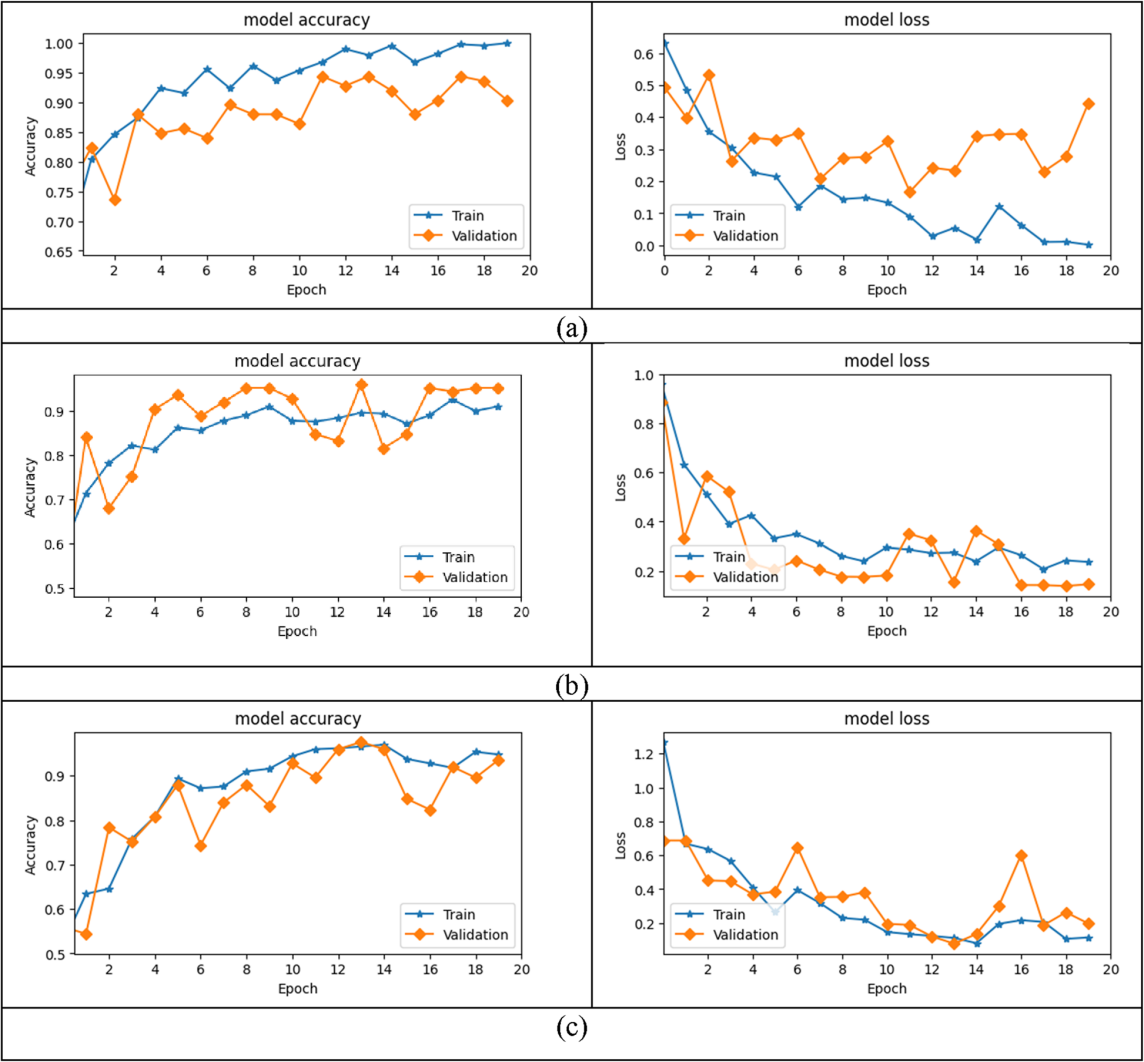
Training and validation process namely model accuracy and loss curves for the CNN models for tuberculosis classification studied in the proposed work. **a** AlexNet (**b**) Shallow-CNN (**c**) DenseNet

We considered a probabilistic model, a Gaussian Process, for the objective function using the Bayesian technique. The objective function is chosen to be the loss function of the shallow-CNN model, and various model parameters are used for optimal performance. The final optimal parameters derived from the preceding approach are listed in Table 2. The Bayesian tuning process, as depicted in Fig. 5, involves the experimentation of various configurations during each trial run. In order to determine the optimal trial run, the validation accuracy is utilised as a criterion for scoring. The S-CNN model configuration parameters utilised in this study were derived from the experimental trial-7 and are presented in Table 2. Figure 6(b) illustrates the S-CNN model's accuracy and loss curves for tuberculosis classification with the optimized parameters obtained from the Bayesian optimization procedure discussed prior. We have also experimented with AlexNet for TB classification and its training procedure is illustrated in Fig. 6(a).

**Table 2:** The tuned Shallow-CNN Structure for Interpretable Tuberculosis Detection (learning rate=0.001, Optimizer = Adam)

**Layer Type**	**Output Shape**	**Number of Kernel**	**Kernel Size**	**Stride**	**Activation**
Input Image	224×224×3	-	-	-	-
Convolution-2D-1	224×224×32	32	5×5	1×1	ReLU
MaxPooling-1	112×112×32	-	3×3	1×1	-
Convolution-2D-2	112×112×64	64	3×3	1×1	ReLU
MaxPooling-2	56×56×64	-	3×3	3×3	-
Convolution-2D-3	56×56×96	96	3×3	1×1	ReLU
MaxPooling-3	28×28×96	-	3×3	3×3	-
Convolution-2D-4	28×28×96	96	3×3	1×1	ReLU
MaxPooling-4	14×14×96	-	3×3	3×3	-
Dense-1	1×512	-	-	-	ReLU
Dense-2	1×2	-	-	-	SoftMax

### DenseNet

DenseNet is a cutting-edge deep neural network that outperformed standard deep learning models in terms of classification accuracy and performance. Through the transfer learning approach, it was also verified for medical picture classification. The performance of the Chest X-ray in the categorization of tuberculosis has been thoroughly researched. DenseNet is notable for its ability to address the vanish gradient problem, allow feature propagation, and lower the model's necessary parameters. To compare the proposed shallow-CNN model, we adopt the DenseNet architecture and tweak the top layers, namely Global Average Pooling (GAP) and a dense layer, to make them acceptable for TB classification. The top layers are retrained for Chest X-rays in order to learn the TB symptoms, while the core dense layer structure and weights are kept [32, 33]. Figure 6(c) illustrate the training and validation process of the DenseNet architecture which would be compared with the S-CNN model for performance analysis.

### Class Activation Map (CAM)

Deep neural nets, which are known to generate strong classification performance, frequently have low transparency and explainability, which is an extensively studied characteristic of deep learning models. Class activation maps are a means of explaining how deep learning models conduct picture classification discrimination. CAM's job is to provide high-resolution visualization of regions of interest in an input image that were used to make a decision. This is very valuable for users like TB radiologists who want to analyze the TB symptoms in the input radiograph to help with disease management. CAM is accomplished by taking a DL model and conducting global average pooling of the penultimate convolution layer. Then, for the improved model, the pooled features are employed as a fully connected layer. The net's weights are then projected back to the convolution layers to produce activation maps that represent the discriminate regions of interest [25, 34]. In this study, we use the CAM to assess the interpretability of shallow-CNN and compare it to DenseNet.

### LIME

Local interpretable model-agnostic explanations (LIME) is an alternate popular explainer system utilized for various machine learning models used for text and image classification. The objective of LIME is to develop a white-box model, namely linear regression, to explain a particular region of an input vector, which is referred to as a locally interpretable system. By creating super-pixels, the LIME system for image classification uses a highly complex trained deep neural model to determine the outcome for different instances of the input sample image. The results of these sampled regions are then weighted based on a measure of similarity. Then, a regressor is fitted locally by minimizing a locality-aware loss function defined as the fidelity function in [35]. In addition to CAM, we use the LIME explainer system to interpret model results for TB detection.

## Experimental results

We attempt to create a shallow-CNN model for TB classification in this paper, so that the network may provide explainable results with dependable performance ratings for diagnosing the disease. With the sample Chest X-ray, the finely tuned proposed model is initially analyzed by generating the filter activation maps for the convolution and maxpooling layers.

**Fig. 7:**
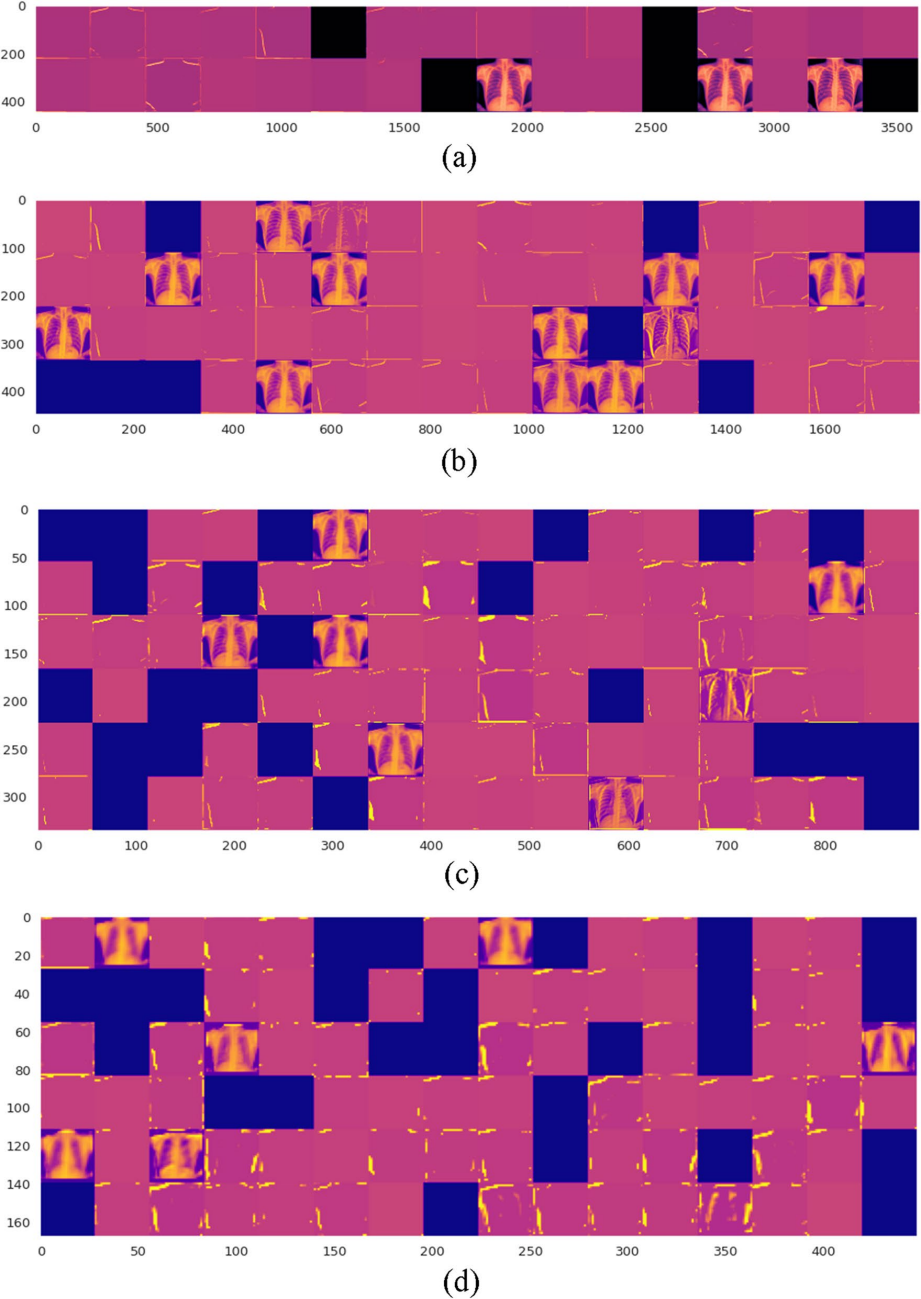
Visualization of the convolutional layers' activation functions for the proposed shallow-CNN model's four layers (**a**-**d**) respectively

The filter activation functions show how the different kernels in the convolution layers respond to different features in the input image. The maxpooling layers then downsample the feature space, resulting in a smaller model with fewer parameters. In Fig. 7(a)-(d), we can see that some of the kernels have activations that are more intense in the abdominal areas of the image. This indicates that these kernels are detecting features that are specific to tuberculosis, such as the presence of nodules or infiltrates in the lungs. The other kernels are responding to other features in the image, such as the edges of the bones and organs.

**Fig. 8:**
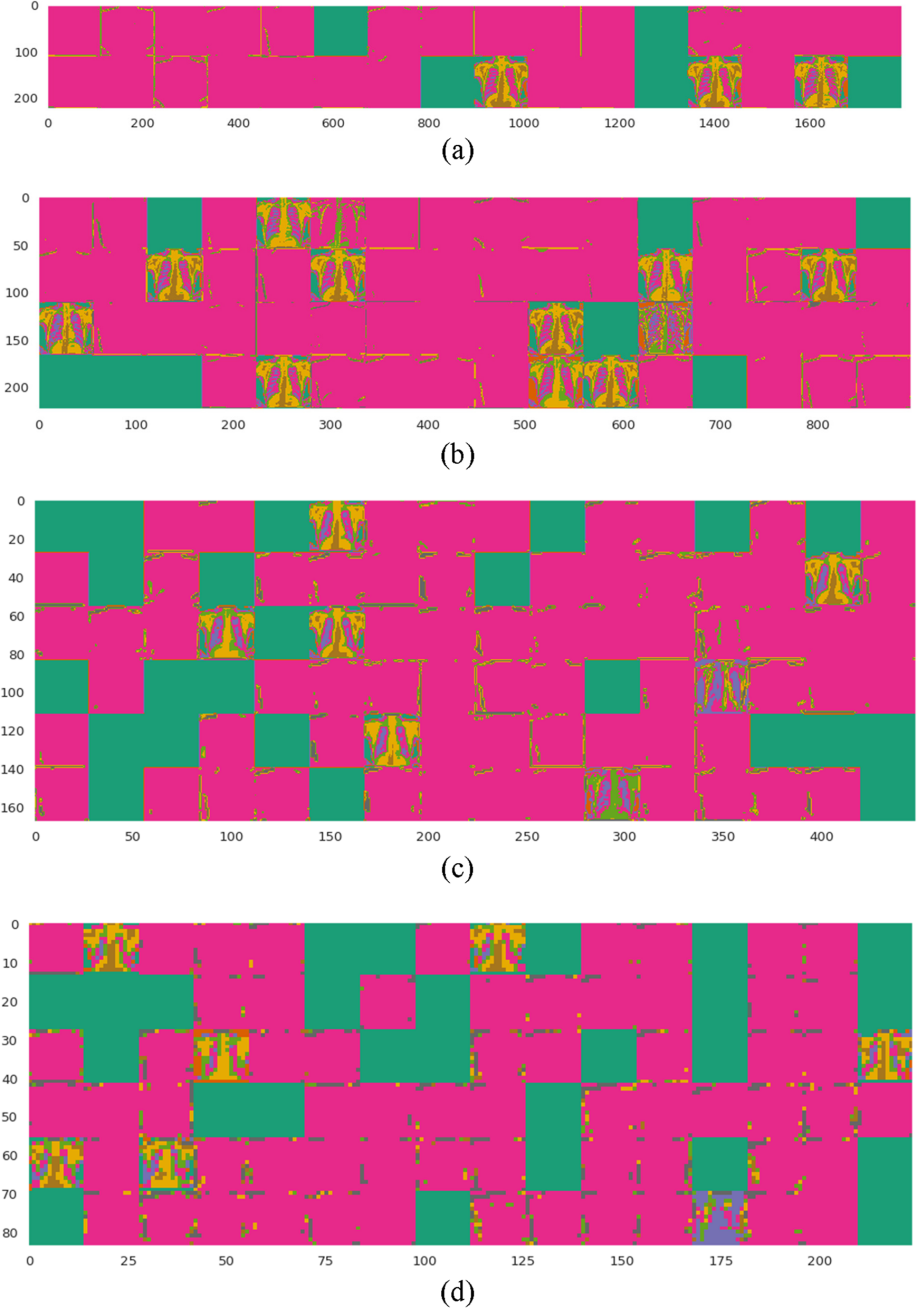
Visualization of the Maxpooling layers' activation functions for the proposed shallow-CNN model's four layers

Figure 8 (a)-(d) shows the weight activation of the maxpooling layers. The weight activation shows how the different pooling windows in the maxpooling layers are responding to the features in the input image. The pooling windows are subsampling the feature space, which reduces the number of parameters in the model. However, the pooling windows are also preserving the most important features in the image, which allows the model to still learn to classify TB with accuracy.

The visualization of the filter activation functions and weight activations can help us to understand how the shallow-CNN model is learning to classify TB. The filter activation functions show us which features the model is paying attention to, and the weight activation functions show us how the model is subsampling the feature space. This information can be used to improve the performance of the model, or to develop new models for TB classification.

**Fig. 9:**
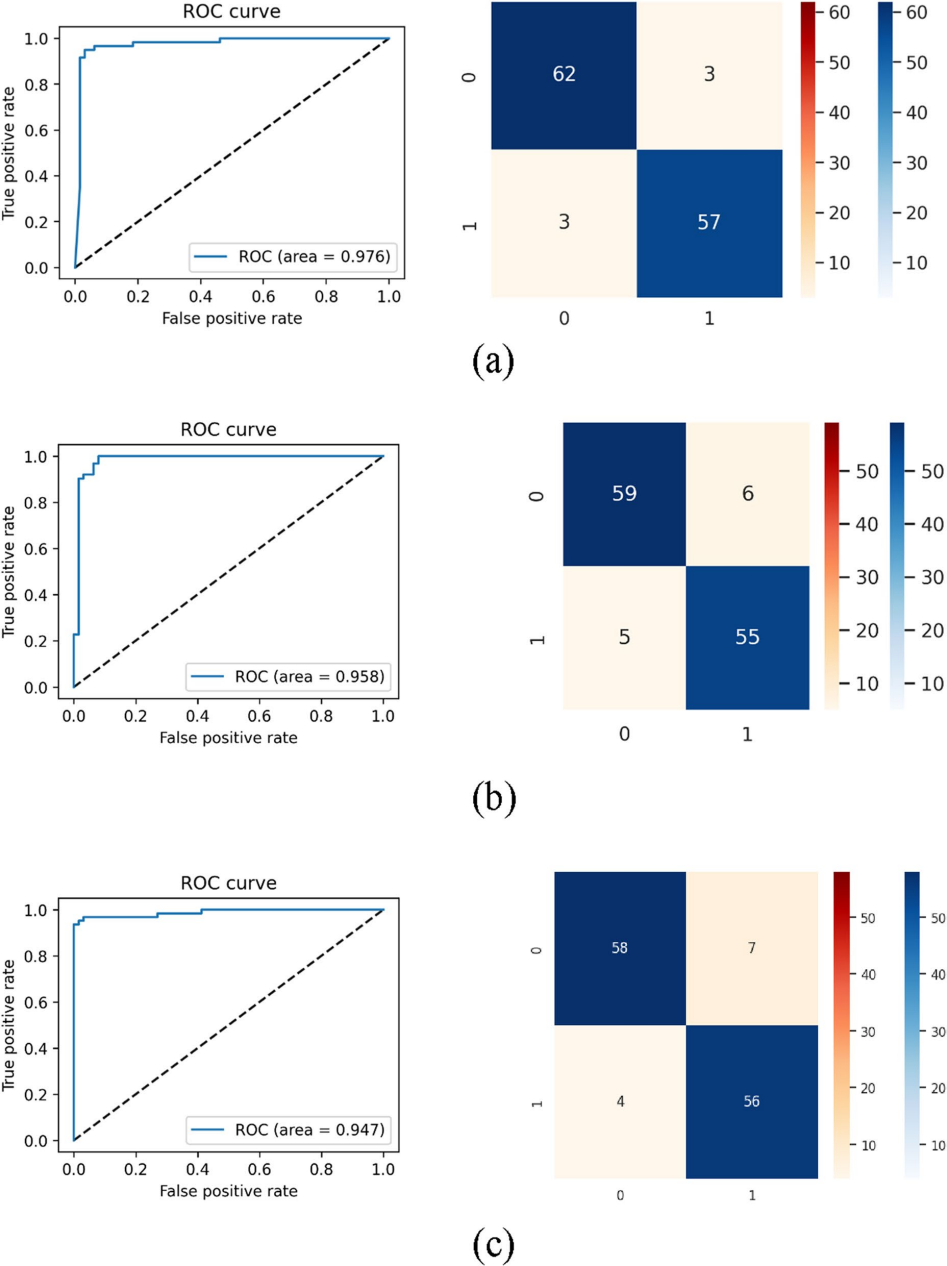
CNN models classification performance for TB detection in terms of Receiver Operating Characteristic (ROC) curve and Confusion matrix (**a**) S-CNN (**b**) DenseNet (**c**) AlexNet

We use the test dataset to determine the performance scores of the proposed shallow-CNN and other models for classification. Figure 9 (a) illustrates the ROC plot's area under the curve, which has a peak value of 0.976, indicating strong classification performance for S-CNN. Furthermore, the proposed model's confusion matrix given in Fig. 9(a) exhibit fewer erroneous predictions (off-diagonal elements), when compared to Fig. 9(b) and (c) which indicates the performance of the DenseNet and AlexNet models for the test dataset respectively.

In order to validate and compare the proposed model with a state-of-the-art network, we have computed the classification scores commonly used in the literature for the modified DenseNet and AlexNet structure with test dataset and tabulated in Tables 3, 4 and 5 respectively.

**Table 3:** Results of the deep learning models considered in this work

**Classification**	**TP**	**FN**	**TN**	**FP**
Modified DenseNet121	55	5	59	6
Shallow-CNN	57	3	62	3
AlexNet	56	4	58	7

**Table 4:** Performance evaluation based on Classification metrics-I

**Classification**	**ACC**	**PRE**	**SEN**	**SPE**	**NPV**
Modified DenseNet121	0.91	0.90	0.92	0.91	0.92
Shallow-CNN	0.95	0.95	0.95	0.95	0.95
AlexNet	0.91	0.89	0.93	0.89	0.94

**Table 5:** Performance evaluation based on Classification metrics-II

Classification	F1-Score	MCC	FDR	FOR
Modified DenseNet121	0.91	0.82	0.098	0.078
Shallow-CNN	0.95	0.90	0.050	0.046
AlexNet	0.91	0.83	0.111	0.064

Table 3 shows test predictions for the three models considered in this work. TP-True Positive, TN-True Negative, FN-False Negative and FP- False Positive of Shallow-CNN provide less wrong predictions compared to the modified DenseNet and AlexNet.

Table 4 shows the classification metrics-I for assessing the performance of the models by means of ACC-Accuracy, PRE-Precision, SEN-Sensitivity, SPE-specificity and NPV-Negative Predictive Value. The proposed Shallow-CNN provide improved scores compared to modified DenseNet and AlexNet for TB classification.

Table 5 shows classification metrics-II for assessing the performance of the models through F1-Score, MCC-Matthews correlation coefficient, FDR-False Discovery Rate, and FOR- False Omission Rate. The metrics verify the ability of the shallow-CNN to perform comparable classification performance with that of DenseNet and AlexNet.

Class activation maps (CAMs) and local interpretable model-agnostic explanations (LIME) are two explainable AI (XAI) methods that can be used to explain the predictions of a deep learning model for TB classification. CAMs and LIME generate heatmaps for each image that highlight the regions (red regions) of the image that are most important for the model's prediction. By analyzing these heatmaps, we can gain a better understanding of how the model is making its predictions and identify potential areas for improvement.

**Fig. 10:**
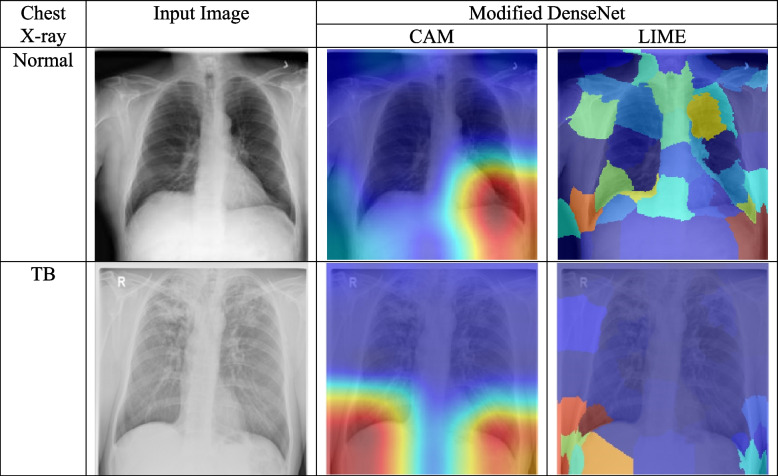
The modified DenseNet model was evaluated using two explainable AI frameworks, CAM and LIME. CAM and LIME was used to generate heatmaps that show the dominant activation regions (red regions) for normal and TB images. The heatmaps showed that the model did not fully learn the features of the lung regions for both normal and TB images

**Fig. 11:**
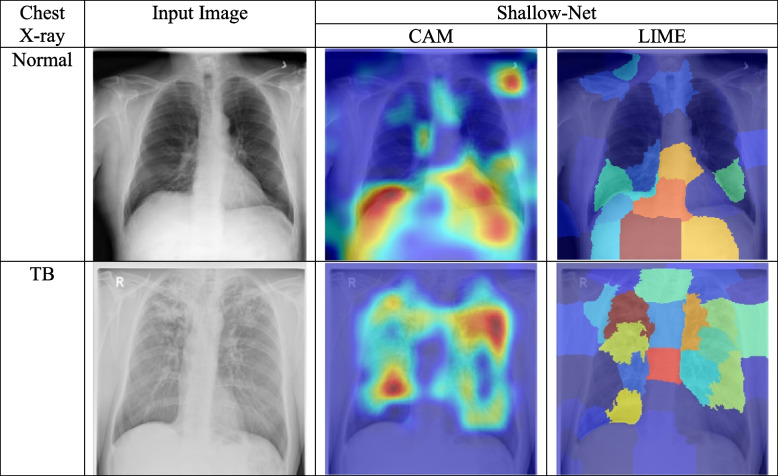
The proposed Shallow-Net model was evaluated using two explainable AI frameworks, CAM and LIME. The heatmaps showed that the model tried to learn the features of the lung regions specifically for both normal and TB images

In the case of the modified DenseNet model shown in Fig. 10, the CAM and LIME heatmaps for both normal and TB images show that the model is predominantly paying attention to features outside of the lung regions. This suggests that the model is not fully learning the features that are most indicative of TB. In contrast, the CAM and LIME heatmaps for the proposed Shallow-Net model shown in Fig. 11 shows that the model is paying more attention to the lung regions (red regions), which is more consistent with the features that are known to be indicative of TB. These results suggest that the proposed Shallow-Net model is better able to learn the features that are important for TB classification than the modified DenseNet model. This is likely due to the fact that the Shallow-Net model is simpler and has fewer parameters, which makes it easier to interpret and troubleshoot.

Overall, the results of this study demonstrate the utility of XAI methods for understanding and improving the performance of deep learning models for TB classification. By analyzing the heatmaps generated by CAM and LIME, we can gain a better understanding of how the model is making its predictions and identify potential areas for improvement. This information can be used to improve the accuracy of the model and to make it more interpretable, which is essential for clinical applications.

## Discussion

In this study, we investigated the efficacy of a shallow Convolutional Neural Network (CNN) architecture, featuring a reduced number of convolution and maxpooling layers, for discerning tuberculosis manifestations within X-ray images. Our primary premise revolves around the feasibility of employing a compact set of convolutional or feature extraction layers to effectively address straightforward image classification tasks, specifically the development of a tuberculosis screening system. Notably, a significant portion of existing literature in this domain predominantly leans towards deeper convolutional neural networks, particularly pre-trained models [6, 7, 19].

Table 6 serves to summarize the relevant body of work concerning tuberculosis classification. However, a direct comparison between these studies is somewhat limited due to the variations in the composition of normal and tuberculosis classes across the works listed. The majority of prior research in TB diagnosis emphasizes the demand for high-accuracy CNN classifiers in the context of X-ray image classification for tuberculosis detection. A case in point is the work by Liu et al., where they formulated a robust CNN classifier leveraging imbalanced TB cases, achieving an accuracy of 85.68% [14]. It's worth noting that their study did not delve into the interpretability of the classifier's decisions. Similarly, Liu and Huang identified DenseNet-121 as a promising classifier for TB diagnosis, boasting a peak accuracy of 0.835. Nevertheless, they omitted an exploration of the inner mechanisms of the DenseNet model concerning TB interpretability [15]. Furthermore, Tasci et al. explored various pre-trained neural networks, including InceptionV3 and Xception, achieving an impressive peak accuracy of 97.699% [19]. However, these sophisticated CNN models, while excelling in classification performance, are challenged by limited explainability and interpretability [15].

**Table 6:** Summary of relevant studies involving tuberculosis classification for comparative analysis

Author	Methodology	Performance Scores
Liu et al. [14]	Convolution Neural Network	Acc: 85.68%
Liu and Huang [15]	Convolution Neural Network	Acc: 0.835
Rahman et al. [6]	Combinational of pre-trained CNN architecture	Acc: 96.47% (unsegmented radiographs)
Tasci et al. [19]	InceptionV3 and Xception CNN	Acc: 97.5% (MC Dataset)
Proposed work	Shallow-CNN	Acc: 95%

Rahman et al.'s work pursued a comprehensive TB diagnosis inclusive of explanations, ultimately favoring DenseNet-201 as the optimal performer with a classification accuracy of 98.6%, leveraging Class Activation Maps (CAM) for TB interpretations [6]. Despite DenseNet's commendable classification and explanation capabilities, its substantial 201-layer structure raises computational complexity concerns associated with training and inference.

In light of this background, our investigation was motivated by the question of whether a more straightforward CNN architecture, characterized by fewer layers and reduced computational complexity, could offer sufficient efficacy in TB screening. Consequently, our inquiry led to the development of a Shallow Convolutional Neural Network (S-CNN). Employing extensive parameter tuning, facilitated by Bayesian optimization, we attained robust classification performance in TB diagnosis from chest X-ray images. The achieved results, including a peak F1-score of 0.95 and an MCC value of 0.9, surpassed those obtained with the modified DenseNet utilized for comparative analysis in this study.

In addition to the comparison with DenseNet, we extended our evaluation to include the AlexNet model, an early deep-layer CNN architecture acclaimed for its outstanding classification accuracy in the ImageNet competition. It is noteworthy, however, that AlexNet was originally designed for the classification of general image sets, in contrast to our purposeful design of the S-CNN model for the specific task of lightweight TB classification. Our findings conclusively demonstrate that the proposed Shallow-CNN, despite its streamlined feature extraction layers, not only rivals but outperforms its deeper counterparts in the context of TB screening. Furthermore, our approach adeptly captures crucial disease symptoms from distinct lung regions for accurate TB diagnosis, as revealed through the utilization of class activation maps and LIME techniques.

However, it's crucial to acknowledge the limitations of our proposed work. Our experimental dataset encompassed a relatively limited number of X-ray images for test purposes, warranting a more comprehensive TB dataset for robust clinical application. Furthermore, this study focuses exclusively on the screening of TB manifestations, necessitating confirmation by a radiologist for precise clinical interpretations.

## Conclusion

In this research paper, we sought to answer the intriguing question of whether a simple CNN architecture with fewer convolution layers would suffice for the pre-screening of tuberculosis from Chest X-rays. The motivation for developing shallow-CNN stems from the fact that it requires fewer computation steps due to a smaller number of trainable parameters and provides straightforward interpretation capabilities.

In this study, we have established a shallow-CNN architecture for accurate classification of TB illness using chest Chest X-rays. We have reported a strong classification performance with a peak accuracy, and F1-score of 0.95 respectively, which is comparable to a state-of-the-art pre-trained deep learning model employed in the study which produced an accuracy and F1-score of 0.91. In terms of MCC, the proposed shallow-CNN produced a maximum value of 0.90, while the modified DenseNet produced 0.82. Consequently, demonstrating that shallow-CNN provides comparable TB screening performance to DenseNet.

The Shallow-Net model is simpler and has fewer parameters than the modified DenseNet model. This makes it easier for the Shallow-Net model to learn the features that are important for TB classification. This also means that the Shallow-Net model is less sensitive to the noise in the training data, which makes it more robust and easier to modify the architecture. Overall, the Shallow-Net model is a better choice for TB classification than the modified DenseNet model because it is simpler, has fewer parameters, and is easier to interpret.

In addition to evaluating the classification performance of the models, class activation maps (CAM) and LIME were used to investigate the explainability of the models in order to facilitate the interpretation of the models for TB diagnosis. The CAM and LIME feature importance maps of the proposed shallow-CNN model provided the necessary rationale for identifying the relevant lung regions, namely the lower lung regions, for TB classification. Thus, we demonstrated that using the suggested shallow-CNN model to screen radiographs for tuberculosis could be advantageous for rapid and interpretable disease management.

## Data Availability

The dataset that support the findings of this study are available in IEEE Dataport with the identifier https://dx.doi.org/10.1109/ACCESS.2020.3031384, 10.21227/mps8-kb56.

## References

[1] Feleke BE, Feleke TE, Biadglegne F (2019). Nutritional status of tuberculosis patients, a comparative cross-sectional study. BMC Pulm Med.

[2] ter Beek L, Bolhuis MS, Jager-Wittenaar H, Brijan RXD, Sturkenboom MGG, Kerstjens HAM (2021). Malnutrition assessment methods in adult patients with tuberculosis: a systematic review. BMJ Open.

[3] Tuberculosis. Who.int.

[4] CDCTB. Tuberculosis. Centers for Disease Control and Prevention. 2022.

[5] Ueda D, Yamamoto A, Shimazaki A, Walston SL, Matsumoto T, Izumi N (2021). Artificial intelligence-supported lung cancer detection by multi-institutional readers with multi-vendor chest radiographs: a retrospective clinical validation study. BMC Cancer.

[6] Rahman T, Khandakar A, Kadir MA, Islam KR, Islam KF, Mazhar R (2020). Reliable tuberculosis detection using chest x-ray with deep learning segmentation and visualization. IEEE Access.

[7] Tang YX, Tang YB, Peng Y, Yan K, Bagheri M, Redd BA, et al. Automated abnormality classification of chest radiographs using deep convolutional neural networks. NPJ Digit Med. 2020. 10.1038/s41746-020-0273-z. 10.1038/s41746-020-0273-z PMC7224391 32435698

[8] Schroeder JD, Bigolin Lanfredi R, Li T, Chan J, Vachet C, Paine R, et al. Prediction of Obstructive Lung Disease from Chest Radiographs via Deep Learning Trained on Pulmonary Function Data. Int J Chron Obstruct Pulmon Dis. 2020. 10.2147/COPD.S279850. 10.2147/COPD.S279850 PMC7801924 33447023

[9] Alhudhaif A, Polat K, Karaman O. Determination of COVID-19 pneumonia based on generalized convolutional neural network model from chest X-ray images. Expert Syst Appl. 2021. 10.1016/j.eswa.2021.115141. 10.1016/j.eswa.2021.115141 PMC8093008 33967405

[10] Saleem HN, Sheikh UU, Abd. Khalid S. Classification of chest diseases from x-ray images on the chexpert dataset. In: Lecture Notes in Electrical Engineering. 2021.

[11] Blumenfeld A, Greenspan H, Konen E, Mori K, Petrick N (2018). Pneumothorax detection in chest radiographs using convolutional neural networks. Medical Imaging 2018: Computer-Aided Diagnosis.

[12] Li X, Xiong H, Li X, Wu X, Zhang X, Liu J (2022). Interpretable deep learning: interpretation, interpretability, trustworthiness, and beyond. Knowl Inf Syst.

[13] Lei F, Liu X, Dai Q, Ling BW-K. Shallow convolutional neural network for image classification. SN Appl Sci. 2020;2:97.

[14] Liu C, Cao Y, Alcantara M, Liu B, Brunette M, Peinado J, et al. TX-CNN: Detecting tuberculosis in chest X-ray images using convolutional neural network. In: 2017 IEEE International Conference on Image Processing (ICIP). IEEE; 2017. p. 2314–8.

[15] Liu J, Huang Y (2020). Comparison of different CNN models in tuberculosis detecting. KSII Trans Internet Inf Syst.

[16] Dinesh Jackson Samuel R, Rajesh Kanna B. Tuberculosis (TB) detection system using deep neural networks. Neural Comput Appl. 2018 315. 2018;31:1533–45.

[17] Kuok CP, Horng MH, Liao YM, Chow NH, Sun YN (2019). An effective and accurate identification system of Mycobacterium tuberculosis using convolution neural networks. Microsc Res Tech.

[18] Bharati S, Podder P, Mondal MRH (2020). Hybrid deep learning for detecting lung diseases from X-ray images. Informatics Med Unlock.

[19] Tasci E, Uluturk C, Ugur A (2021). A voting-based ensemble deep learning method focusing on image augmentation and preprocessing variations for tuberculosis detection. Neural Comput Appl.

[20] Ahmed IA, Senan EM, Shatnawi HSA, Alkhraisha ZM, Al-Azzam MMA (2023). Multi-techniques for analyzing x-ray images for early detection and differentiation of pneumonia and tuberculosis based on hybrid features. Diagnostics.

[21] Iqbal A, Usman M, Ahmed Z (2022). An efficient deep learning-based framework for tuberculosis detection using chest X-ray images. Tuberculosis.

[22] Chandra TB, Verma K, Singh BK, Jain D, Netam SS (2020). Automatic detection of tuberculosis related abnormalities in Chest X-ray images using hierarchical feature extraction scheme. Expert Syst Appl.

[23] van der Velden BHM, Kuijf HJ, Gilhuijs KGA, Viergever MA (2022). Explainable artificial intelligence (XAI) in deep learning-based medical image analysis. Med Image Anal.

[24] Kim D, Chung J, Choi J, Succi MD, Conklin J, Longo MGF (2022). Accurate auto-labeling of chest X-ray images based on quantitative similarity to an explainable AI model. Nat Commun.

[25] Zhou B, Khosla A, Lapedriza A, Oliva A, Torralba A. Learning Deep Features for Discriminative Localization. In: Proceedings of the IEEE Computer Society Conference on Computer Vision and Pattern Recognition. 2016.

[26] Magesh PR, Myloth RD, Tom RJ (2020). An explainable machine learning model for early detection of Parkinson’s Disease using LIME on DaTSCAN imagery. Comput Biol Med.

[27] Rahman T, Khandakar A, Chowdhury MEH. Tuberculosis (TB) chest X-ray database | IEEE DataPort. 2020.

[28] Krizhevsky A, Sutskever I, Hinton GE (2017). ImageNet classification with deep convolutional neural networks. Commun ACM.

[29] Mukherjee H, Ghosh S, Dhar A, Obaidullah SM, Santosh KC, Roy K (2021). Shallow convolutional neural network for COVID-19 outbreak screening using chest x-rays. Cognit Comput.

[30] Victoria AH, Maragatham G (2021). Automatic tuning of hyperparameters using Bayesian optimization. Evol Syst.

[31] Cho H, Kim Y, Lee E, Choi D, Lee Y, Rhee W (2020). Basic enhancement strategies when using bayesian optimization for hyperparameter tuning of deep neural networks. IEEE Access.

[32] Huang G, Liu Z, Van Der Maaten L, Weinberger KQ. Densely connected convolutional networks. In: Proceedings - 30th IEEE Conference on Computer Vision and Pattern Recognition, CVPR 2017. 2017.

[33] Zhang K, Guo Y, Wang X, Yuan J, Ding Q (2019). Multiple feature reweight DenseNet for image classification. IEEE Access.

[34] Selvaraju RR, Cogswell M, Das A, Vedantam R, Parikh D, Batra D (2020). Grad-CAM: visual explanations from deep networks via gradient-based localization. Int J Comput Vis.

[35] Ribeiro MT, Singh S, Guestrin C. “Why Should I Trust You?”: Explaining the Predictions of Any Classifier. 2016.

